# Editorial: Central Cardiovascular and Respiratory Control: New Techniques, New Directions, New Horizons

**DOI:** 10.3389/fphys.2020.617092

**Published:** 2020-11-19

**Authors:** Vaughan G. Macefield, Geoffrey A. Head

**Affiliations:** Human Autonomic Neurophysiology Laboratory and Neuropharmacology Laboratory, Baker Heart and Diabetes Institute, Melbourne, VIC, Australia

**Keywords:** sympathetic outflow, connectome, heart failure, hypertension, respiratory regulation, cardiovascular control, respiratory rhythmogenesis

## Introduction

How the brain controls blood pressure and respiration has fascinated physiologists for over a century. The cardiovascular and respiratory regulatory systems are tightly coupled, operating within a narrow range to maintain blood pressure, O_2_, and CO_2_ relatively constant, but with the capacity to operate at different levels according to behavioral or environmental requirements. For a long time, we have known that a decerebrate animal can breathe and maintain its blood pressure with only the brainstem intact. We have learnt much about the circuitry within the medulla required for the beat-to-beat control of blood pressure and heart rate, and the circuitry within the medulla and pons required for the generation of tidal breathing. We have also learnt about the roles of other subcortical structures, such as the hypothalamus and amygdala, in homeostatic regulation of blood pressure and respiration, and the contributions of cortical structures to this control. Much of this knowledge has come through the development of novel techniques, such as viral tracing of pathways, use of the working-heart brainstem preparation and, most recently, optogenetics. New directions that have also changed our focus relate in particular to the genetic and “omics” revolution which has transformed biomedical research in recent years. New models with tissue-specific over-expression, constitutively active receptors and dominant negative genetics have been developed and continue to increase our physiological understanding of these complex systems regulating blood pressure and respiration and how they change during states of disease. There are also new technologies such as CRISPR/Cas9 to enable global, conditional and targeted gene editing. The discoveries of the exquisite detail involved in intracellular signaling, second messengers and biochemical pathways as well as post-transcriptional modulation have markedly enhanced our understanding of regulatory influences and have increased our understanding of physiological processes to a new height.

It is clear that physiologists and pharmacologists will need to be able to integrate a vast array of information to be able to translate preclinical to clinical phases of discovery. Progress continues to be made in identifying the functional connectomes responsible for cardiovascular and respiratory control, and we are now at the stage where some of the work conducted in anesthetized or conscious animals is being pursued in humans through brain imaging. But where does the future lie? What research should we be doing, how should we do it and where will that lead? This Research Topic builds on an annual meeting held in Australia for over 25 years, in which neuroscientists meet to discuss their latest work in central cardiovascular and respiratory control. We have 14 excellent papers in the Research Topic which includes 6 reviews and 8 original articles. Of these, 5 were published in the Frontiers in Neuroscience section while the remaining 9 were published in Frontiers in Physiology and have been viewed over 50,000 times so far. One of the reviews, by Saleeba et al. and Saleeba et al., provides an accessible “how-to guide” to all the approaches that have been used to identify functional networks within the brain. Geared to the student, but also suitable for the avid experimentalist, this article has been hugely popular. Given that it has been viewed >27,000 times it is safe to say that these approaches continue to intrigue and to offer unprecedented access to the networks of the brain. Needless to say, new techniques continue to be developed, and novel applications for their use will continue to allow progress in studying the pathways responsible for cardiovascular and respiratory control.

## New Directions in Human Autonomic Research

A number of the contributions highlighted new and sophisticated techniques for analyzing autonomic function in humans. For example, a novel means of assessing sympathetic outflow non-invasively in humans has been proposed by Jendzjowsky et al.. Using optical coherence tomography, a means of imaging the different layers of the retina as well as the overlying blood vessels, the authors show that choroid vascular perfusion density (VPD) correlates very well with muscle sympathetic nerve activity (MSNA), recorded via an intraneural microelectrode: the higher the MSNA the lower the VPD (Jendzjowsky et al.). Interestingly, there was no relationship between MSNA and retinal VPD, suggesting the latter is not under sympathetic control, while choroid VPD decreased during the cold-pressor response and during an apnoea, maneuvers known to increase MSNA. Importantly, this means that imaging the eye allows one to examine sympathetic vasoconstrictor drive and vascular reactivity together and non-invasively. Another human imaging study, in which functional magnetic resonance imaging (fMRI) of the brain is performed at the same time as recording MSNA has revealed the functional location of the human homolog of the rostral ventrolateral medulla (RVLM), which plays a key role in the generation of MSNA (Macefield and Henderson). This combination of imaging and recording sympathetic activity is unique to these investigators and has provided an amazing insight into the pathways and nuclei involved in regulation of sympathetic activity. However, the technology has been further extended so that Macefield and Henderson have been able to show that changes in activity in this nucleus can be measured through brain imaging, revealing excitatory, and inhibitory mechanisms during sympatho-excitatory maneuvers and extending their work on MSNA-coupled fMRI to uncover operation of the human sympathetic connectome. The interesting aspect of this work is the connections from the prefrontal cortex highlight the importance of the cortex and perception of the environment. On that theme (Faull et al.) found the perception of the exertion involved in exercise was influenced by a state of ketoacidosis. Interestingly, the rating of perceived exertion was more influenced by localized perception of breathlessness and discomfort in the exercising legs rather than the presence of acidosis, but overall suggests that there is a complex interplay of humoral signals which may have evolved to cope with exertion under times of extreme metabolic stress. The considerable advancement in precise imaging coupled with neural recording and stimulation technology for studying human physiology are now enabling us to gain a new insight into the complexities of autonomic function.

## New Directions in Pre-Clinical Autonomic Research

In the area of basic autonomic research, a number of contributions to the topic have included technically sophisticated and cutting-edge technology. Abukar et al. examined a novel micro embolism model of heart failure in sheep to determine the extent of any changes to neural control as well to determine whether this model reflected human heart failure. The technique involves using three sequential embolizations over 3 weeks and to study the animals 12–14 weeks later when heart failure was evident, as indicated by elevated left ventricular end-diastolic pressure (Abukar et al.). Assessment of neural control involved examining the heart rate and blood pressure baroreflex relationship, which was markedly reduced in the heart failure group compared to the control animals. Importantly, these findings and other aspects of the model in generating increased respiratory rate and incidence of apnoea suggests that this large animal model of heart failure replicates the neural and respiratory instabilities seen in the human form of the disease. Zhang et al., using an interesting model of volume overload in rats, tested the influence of the sympathetic nervous system using bilateral stellate ganglionectomy and found significant improvement in cardiac fibrosis associated with decreased cardiac norepinephrine and TGF beta-1 release. Dhingra et al. explore the critical brainstem nuclei involved in respiratory rhythmogenesis, and the contributions of neighboring nuclei in furnishing the normal respiratory pattern through an elegant series of experiments conducted in the working heart-brainstem preparation. They demonstrated that balance of excitation and inhibition exerted onto the central network determines the overall respiratory pattern. In terms of sensory feedback, Driessen presents novel data on the central projections of afferents traveling in the trigeminal, vagus, glossopharyngeal, and spinal nerves, and the role of a hitherto largely unknown nucleus in the lateral medulla, the paratrigeminal nucleus, in multimodal processing of visceral sensory information and its integration for respiratory and cardiovascular control. Continuing on the theme of respiration, and its interaction with cardiovascular control, using the acute intermittent hypoxia (AIH) model, Farnham et al. argue that a specific excitatory amino acid, PACAP, is responsible for the sympatho-excitation resulting from AIH, as shown by direct injection of PACAP into the RVLM. They suggest this may be involved in the hypertension associated with obstructive sleep apnoea. Dereli et al. identify several brainstem sites in which long-term hypercapnia leads to blunted chemosensitivity, offsetting the increase in ventilation that results from elevated CO_2_ levels, with the neuropeptide galanin playing a key role.

There is increasing interest in non-drug therapies for the treatment of hypertension. A quite novel approach has been to use electroacupuncture, which appears to be quite effective in producing a sustained decrease in blood pressure (Li et al., 2015). However, the mechanisms aren't well-understood. Malik et al. examined whether adenosine in the rostral ventrolateral medulla contributes to electroacupuncture modulation of sympatho-excitatory reflexes through an adenosine receptor-opioid mechanism. These elegant studies led to the conclusion that activation of opioid receptors in this region of the medulla inhibits pre-sympathetic activity directly as well through an adenosine related mechanism, resulting in a lowering of blood pressure. Moreover, we now know that the central cardiovascular networks can be affected by inflammatory mediators, with Elsaafien et al. showing central as well as peripheral upregulation of several inflammatory markers in hypertension, arguing that it is the central attraction of these agents to key autonomic nuclei that lead to the increase in sympathetic outflow in high blood pressure.

## Latest Contributions by Specific Topic Review

A comprehensive review by Badoer provided an insight into the contribution of the carotid body in cardiovascular and metabolic dysfunction. In particular the concept that overactivity of the carotid body may contribute to both hypertension as well as greater sympathetic activity in metabolic disturbances associated with obesity and insulin resistance. This is an exciting new area since targeting the carotid body for ablation may be a viable treatment for essential as well as obesity- induced hypertension. There is also the possibility of modulating the activity of the carotid body afferents by targeting specific receptors such as P2X3 or ion channels such as TRPM7. To date this treatment has been successful in small clinical studies in drug resistant hypertension and in patients with low ejection fraction heart failure. Importantly the procedure appears to be quite safe to perform (Badoer). Jackson et al. provided an up-to-the-minute examination of the mechanisms responsible for genetic hypertension in the Schlager BPH mouse strain which was developed in the 1970s. This is the first comprehensive review of this strain, which has been used extensively as the mouse equivalent of the spontaneous hypertensive rat (SHR) but there are important differences which relate to the contribution of the sympathetic nervous system in the Schlager strain which appears to be the predominant mechanism driving the hypertension (Jackson et al.). The main evidence for this is that ganglion blockade completely abolishes the difference between normotensive and hypertensive mouse strains but this is not the case in SHR. The review also covers important findings relating to a marked contribution of the medial amygdala to the hypertension in BPH mice as well as the susceptibility to attenuate the hypertension with neurosteroids such as allopregnanolone, which upregulate neuro steroid sensitive extrasynaptic GABA A receptors, particularly in the hypothalamus and amygdala (Jackson et al.). Thus, the Schlager hypertensive mouse provides a relatively pure neurogenic model of hypertension but exactly how this relates to human essential hypertension is not exactly known at this stage.

## Conclusion

This collection of papers and reviews provides the reader with an overview of the current state-of-the-art in central cardiovascular and respiratory control. We have highlighted some of the findings from the use of the latest technical advances not only in animal models but also in human autonomic neurophysiology, both of which are helping unravel the complexities of the networks governing some of the most important homeostatic regulatory systems. The importance of these systems is such that they are robust with multiple redundant components but nevertheless can be disturbed, leading to disease. Of course, there is much that we still do not know, so the research will continue. We look forward to receiving submissions for the second volume of this Research Topic.

## Author Contributions

GH and VM contributed to the writing of this editorial. All authors contributed to the article and approved the submitted version.

## Conflict of Interest

The authors declare that the research was conducted in the absence of any commercial or financial relationships that could be construed as a potential conflict of interest.
